# Dental and skeletal findings of 140 wild rabbits (*Oryctolagus cuniculus*) in Finland 2010–2015

**DOI:** 10.1186/s13028-026-00855-8

**Published:** 2026-01-29

**Authors:** Vilma Reunanen, Nelly Jormakka, Johanna Mäkitaipale

**Affiliations:** 1 Department of Equine and Small Animal Medicine, Faculty of Veterinary Medicine, University of Helsinki, P.O. Box 57, Helsinki, FI-00014 Finland; 2 IVC Evidensia Tammisto Animal Hospital, Tammiston Kauppatie 29, Vantaa, FI- 01510 Finland

**Keywords:** Dental disease, Transitional vertebrae, Vertebral column formula

## Abstract

**Background:**

Dental disease is the most common non-infectious disease of domestic rabbits, with a prevalence reaching up to 40% in studied populations. Diet has been shown to be the main cause. Skeletal disorders, such as vertebral column malformations, are also common, affecting 40% of some pet rabbit breeds. Both can lead to severe health issues and decrease the quality of life. We aimed to evaluate the prevalence of dental and skeletal disorders in a Finnish wild rabbit population originating from released domestic rabbits at least four decades ago. As these diseases are related to genetics, diet, and housing in pet rabbits, we hypothesized that prevalence of these diseases is low in wild rabbits.

**Results:**

Physical examination and radiographs (laterolateral *n* = 140, ventrodorsal *n* = 47) were used to study 140 wild rabbits hunted from the Helsinki City area. Mild signs of dental disease (elongation of premolar tooth root) were observed in radiographs of 12.6% of the rabbits. The vertebral formula was C7/Th12/L7/S4 in 89.1% of the rabbits, although five other formulae were also identified. Prevalence of transitional vertebrae was 13.9%. Ankylosing malformations were not identified. Traumatic lesions were found in 15% of the rabbits.

**Conclusions:**

Initial signs of dental disease were identified in a Finnish wild rabbit population, although the prevalence was lower than in previously investigated pet rabbits. More advanced stages of dental disease or vertebral column ankylosing malformations were not identified. Prevalence of vertebral column deformities was low.

## Background

Acquired dental disease is the most common non-infectious disease of domestic rabbits, affecting up to 25–40% of studied populations [1–3]. The aetiology is strongly associated with diet [3–6]. Energy-dense, low-calcium, and high-sugar/starch diets like fruits and ´muesli mixture´ have been suggested to cause less chewing-training and to increase the risk for metabolic bone diseases that reduce alveolar bone density [6]. The high sugar/starch content of these diets causes oral microbiota dysbiosis and plaque formation, posing a risk for periodontitis. Taken together, these cause a loss of alveolar integrity, which leads to malocclusion [6]. The diet of rabbits should be high in forages, fresh or dried hay, and similar high-fibre compound feeds [6]. Wild rabbits’ diet consists mainly of different grass species, grass-forbs, and seedlings, although rabbits also consume cereals and nuts like acorns when available [7–9]. Their diet contains abundant fibre and lacks commercial concentrated rabbit food with artificial compounds and is therefore suggested to be optimal for prevention of dental disease [10]. Mild stages of dental disease (elongation of premolar and molar tooth roots) have, however, been reported in Japanese hares [11]. Thus far, prevalence has not been investigated in wild rabbits.

Congenital vertebral anomalies, specifically transitional vertebrae and hemivertebrae, are common in domestic rabbits [2, 12, 13]. Previously has been reported overall prevalences of 15% and 18.0% and even up to 40% and 57%, respectively, for Dwarf Lop rabbits in Finnish and Czech studies [2, 13]. Degenerative spinal disorders, such as spondylosis, calcified intervertebral discs, narrowed disc spaces, and facet joint arthritis, are common in older pet rabbits, of which 15% were reported to be affected [2]. These spinal disorders are frequently detected in radiographs of asymptomatic rabbits but may potentially cause pain and predispose to urinary disorders, perineal scalding, and pododermatitis [12].

The structure and formula of the vertebral column is highly conserved within mammals, especially within dorsomobile runners [14]. However, a more variable normal vertebral formula has been suggested for domestic rabbits [13, 15]. Transitional vertebrae may occur at the junctions of all vertebral regions and have morphological features of the adjacent vertebral region [16]. A lumbosacral transitional vertebra (LTV) can be classified as lumbarization when the first sacral vertebra has morphological features of a lumbar vertebra or sacralization when the last lumbar vertebra has morphological features of a sacral vertebra [16, 17]. In complete lumbarization, the presacral vertebral count is one higher than the normal and in complete sacralization one lower than the normal vertebral count for the species [18]. Therefore, the entire spine must be radiographed for an exact radiographic evaluation of the vertebral formula and to diagnose transitional vertebrae, including LTV [17]. In addition to rabbits, these congenital vertebral anomalies have been reported in several other species [17, 19–23]. For example, high prevalence of LTVs has been observed in an isolated inbred wolf population [21], whereas hemivertebrae are very common in brachycephalic screw-tailed dog breeds [24]. Moderately high heritability estimates have been reported for LTV in the German shepherd dog breed [25, 26] and for hemivertebrae in brachycephalic screw-tailed dog breeds [27]. These anomalies are less investigated in rabbits, and the aetiology of ankylosing deformities is unclear, although studies suggest strongly that brachycephaly could be associated with hemivertebrae [2, 13]. In breeding does, small cage size predisposed to ankylosing vertebral column deformities [28].

The aim of this descriptive study was to report the prevalence of dental disorders and congenital vertebral anomalies (transitional vertebrae and hemivertebrae) in addition to degenerative skeletal disorders (spondylosis and osteoarthritis) and skeletal injuries (fractures) in our random study population of Finnish wild rabbits. We also aimed to describe the normal vertebral formula in wild rabbits. As these diseases are related to genetics, diet, and housing in pet rabbits, we hypothesized that prevalence of these diseases is low in wild rabbits.

## Methods

### Animals

European rabbit (*Oryctolagus cuniculus*) is classified as an invasive alien species in Finland originating from domesticated rabbit populations released to the Helsinki City area in the 1980s [9]. Our sample population consists of 140 wild rabbits hunted from the Helsinki city area without any selection as a yearly project of culling the wild rabbit population during hunting season (1 September to 31 March) in 2010–2011 (*n* = 93) and 2014–2015 (*n* = 47). Rabbits were stored frozen (– 25 °C) until thawed for this study. Examinations and radiography of 93 rabbits were performed between May 2012 and June 2013 at the University of Helsinki Veterinary Teaching Hospital. Forty-seven rabbits were examined, weighed, and radiographed in October 2023 at IVC Evidensia Tammisto Animal Hospital.

### Physical examination

During the examination, the colour and sex of rabbits were recorded. The skull was palpated for such irregularities as abscesses, tumours, fractures, elongated tooth roots in mandibles, and dislocated mandibles and the extremities for joint dislocations, effusions, fractures, and other irregularities. Incisors were examined for horizontal ribbing, malocclusion, and absence. Molar teeth and oral cavity were examined using an otoscope and abnormalities like spurs, missing teeth, traumatic lesions, infections, and tumours were recorded. Dentition was graded using the previously published grading system of Progressive Syndrome of Acquired Dental Disease (PSADD) [29]. Skin lesions were reported in 47 rabbits examined in October 2023.

### Radiography

Laterolateral skull and in-toto radiographs of all 140 rabbits were taken. Of these 140 rabbits, in-toto ventrodorsal radiographs of 47 rabbits were also taken. Thus, the study population of 140 rabbits consisted of two subpopulations with different sets of radiographs. Subpopulation 1 of 93 rabbits included laterolateral skull and in-toto radiographs of the thorax and abdomen for evaluation of the thoracic and lumbar vertebral column. Subpopulation 2 of 47 rabbits also included also ventrodorsal radiographs of the thorax and abdomen, and additionally laterolateral and vetrodorsal radiographs of the extremities and tail. Due to intra- and post-mortem traumas during handling of carcasses (fractures) and technical and quality errors in radiography, number of rabbits varied in analyses. Intra- and post-mortem traumas were not included in the analyses. Indirect (CPI Indico 100 RAD 150 kV 2006, Fujifilm FCR XG-1) and direct (Agfa DXD40 1000 C) digital imaging techniques were utilized.

### Image analysis

**Fig. 1:**
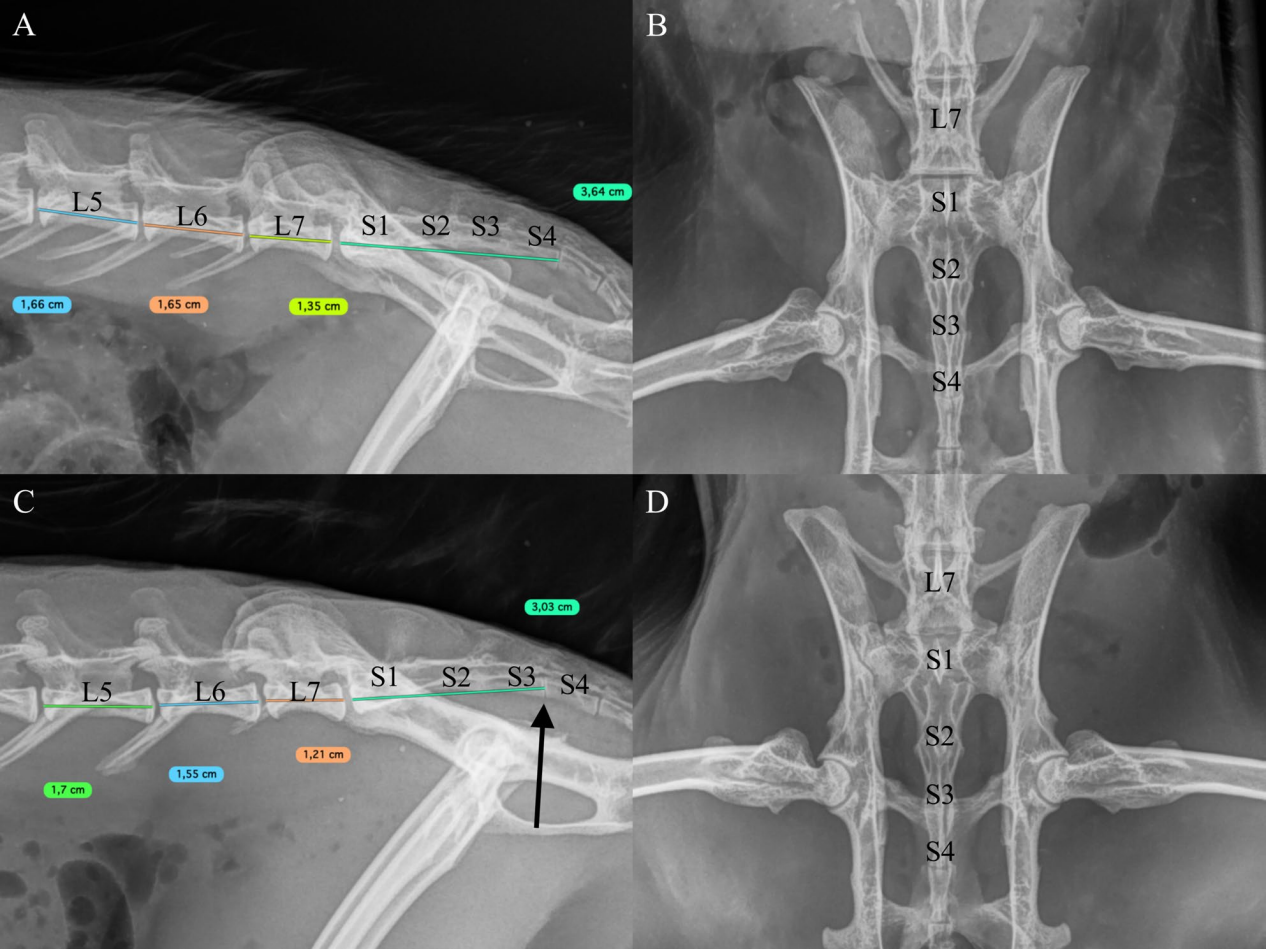
Radiographic evaluation and length measurement of lumbosacral junction in Finnish wild rabbits. *Cd* caudal vertebra, *L* lumbar vertebrae, *LTV* lumbosacral transitional vertebra, *S* sacral vertebra. **A** Laterolateral and **B** ventrodorsal image of the lumbosacral junction of a wild rabbit with normal spinal morphology. **C** Laterolateral and D: ventrodorsal image of lumbosacral junction of a wild rabbit with features resembling mild LTV: Only three sacral vertebrae when the S4 has the morphology of Cd1 (S3-S4 vertebral space marked with arrow). The location of L7 in relation to iliac wings is more caudal, relative length of L7 is mildly shorter, and the transverse processes point more laterally. Length measurement of the L5, L6, L7, and sacrum are performed to assess lumbosacral transitional vertebrae and noted herein

The dentition was graded using the previously published grading system for PSADD [29]. Other findings, such as abnormal calcification, bone lysis, and fractures, were also recorded. In-toto radiographs were evaluated for all visible skeletal abnormalities, including congenital vertebral anomalies, spondylosis, osteoarthritis, discospondylitis, calcified intervertebral discs, narrowed disc spaces, and fractures. The number of cervical, thoracic, lumbar, sacral, and caudal vertebrae was recorded. Transitional vertebrae were then classified [17, 30] according to modified classification based on previous literature [30], Table 1. To evaluate LTV features, the lengths of the L5, L6, and L7 vertebrae as well as the sacrum were measured as presented in Fig. 1. The relative length of L7/L6 was then calculated. In addition, the location of L7 in relation to iliac wings was recorded to support the evaluation of possible LTV [30]. Mild lumbosacral transitional vertebrae (LTV1) based on five (S5) or three (S3) sacral vertebrae were classified as lumbarization or sacralization based on L7/L6 relative length: longer than average length as lumbarization and shorter than average as sacralization. The anticlinal vertebra was also identified [13, 31]. Symmetry of the iliosacral joint and L7 was evaluated. Image analyses were performed separately by all authors, and the results were compared. Diverging results were discussed, and a consensus was achieved.

**Table 1:** Normal vertebral formula and classification of transitional lumbosacral vertebrae with prevalences in Finnish wild rabbits

Formula +/– additional definition (*n*)				
LTV0 (normal): C7/T12/L7/S4 (118, 86.1%)				
LTV grade	Lumbarization		Sacralization	
LTV1 (mild) (15, 10.9%)	C7/T12/L7/S4 + divided sacral middle crest +/– mildly incomplete fusion of S1-S2 (2, 1.5%)	C7/T12/L7/S5 +/– divided sacral middle crest (5, 3.6%)	C7/T12/L7/S4 + L7 short and caudally located (2, 1.5%)	C7/T12/L7/S3 +/– L7 short and caudally located (3, 3.6%)
		C7/T12/L7/S3 +/– divided sacral middle crest (0, 0%)		C7/T12/L7/S5 +/– L7 short and caudally located (3, 3.6%)
LTV2 (symmetrical) (*0–1, 0-0.7%)	C7/T12/L8/S4 + underdeveloped LS space + transitional transverse processes (*0–1, 0–0.7%)		C7/T12/L6/S4 + underdeveloped LS space + transitional transverse processes (0, 0%)	
LTV3 (asymmetrical) (*0–1, 0-0.7%)	C7/T12/L8/S4 + underdeveloped LS space + asymmetrical transitional transverse processes (*0–1, 0–0.7%)		C7/T12/L6/S4 + underdeveloped LS space + transitional transverse processes (0, 0%)	
LTV4 (complete) (3, 2.2%)	C7/T12/L8/S4 (0, 0%)		C7/T12/L6/S4 (1, 0.7%)	
			C7/T12/L6/S5 (2, 1.5%)	

*LTV* transitional lumbosacral vertebra,* C* cervical vertebrae,* T* thoracic vertebrae,* L* lumbar vertebrae, * S* sacral vertebrae. *Ventrodorsal image is missing, thus separation of LTV2 and LTV3 is not possible. LTV grading modified based on Lappalainen et al. [30]. Normal vertebral formula and classification of LTV with the definitions and the observed prevalences in a study population of Finnish wild rabbits (*n* = 137) are presented here

Closure of the physes in the radiographs was recorded for age estimation (proximal and distal femur, proximal and distal tibia, proximal humerus, proximal and distal radius, proximal and distal ulna). Rabbits were divided into three groups depending on the closure of the physes. Rabbits in group one had all visible physes closed (adults), rabbits in group two had at least one radiolucent open physis (juvenile), and rabbits in group three had at least one radiopaque physeal scar visible (young adults). All image analyses were performed during 2024 using DICOM viewer (Pixmeo OsiriX, version 13.0, Bernex, Switzerland and Horos v3.3.6 https://horosproject.org/).

### Statistical analysis

Data analysis and statistics were carried out by using IBM SPSS Statistics 31.0.0. Fisher’s exact and likelihood ratio tests were first used to evaluate the statistical significance of the association between the variables. Descriptive statistics are reported as mean and standard deviation or median and range, depending on the sample distribution. Normality of data distribution was assessed by Kolmogorov-Smirnov test for vertebral length measurements. Mann-Whitney U test was used for analyses with two variables and Kruskall-Wallis H test for analyses with more than two variables. The significance level of P˂0.05 was used for all analyses.

## Results

Out of 140 rabbits, 78 (55.7%) were male and 62 (44.3%) female rabbits. Weight was available for subpopulation 2 (*n* = 47), and mean weight was 1.45 kg (range 0.88–1.87 kg). Most of the rabbits were agouti coloured in different shades of brown (*n* = 129, 92.1%), whereas 11 (7.9%) were black and tan. Nine of the black and tan rabbits were male. Among subpopulation 2 (*n* = 47), skin lesions (wounds) were reported in 14 (29.8%); 11 of these were male rabbits.

**Fig. 2:**
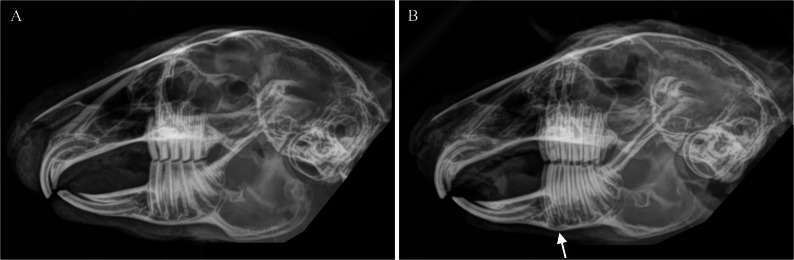
Laterolateral radiographs of two wild rabbits with different dental grades (1 and 2). **A** Laterolateral image of a wild rabbit with normal dentition (dental grade 1). **B** Laterolateral image of a wild rabbit with mild elongation of first premolar tooth rooth (marked with arrow) (dental grade 2)

Seventeen (12.4%) out of 137 rabbits had signs of initial stage of dental disease (mild elongation of the premolar root with thinning of the mandibular bone) in radiographs (grade 2) as presented in Fig. 2B. Five of these were juvenile, two young adults and five adults. Data of age estimation was missing in five cases. In three rabbits, the skull radiograph was excluded due to intra/postmortem fractures of the skull or quality of the radiograph (asymmetry). Only two rabbits with initial stage of dental disease in radiographs (*n* = 17) had mild irregularities in oral examination of one first lower premolar but were graded as normal (grade 1) in radiographs. Abnormalities in the incisor bite were identified in the radiographs of 8 out of 134 rabbits. In all of these cases, bite was not even. Horizontal ribbing, malocclusion, and absence were not observed in the examination of incisors. Mild irregularities in the incisal edge (most often rounding) were common but not registered. No differences were found in prevalence of dental disease between female and male rabbits (*P* > 0.05).

The vertebral formula was C7/Th12/L7/S4 in 122 (89.1%) out of 137 rabbits. LTV were observed in 19 (13.9%) out of 137 rabbits and classification of LTVs with respective prevalences in our study population are presented in Table 1. We did not distinguish if one rabbit had symmetric (LTV2) or asymmetric LTVs (LTV3), because of the lack of ventrodorsal radiograph, but we did not detect abnormal transverse process on the laterolateral view.

Number of presacral vertebrae was 26 in 95 rabbits(*n* = 98, 97%) and 25 in three rabbits (3%). Seven cervical vertebrae were identified in all 98 rabbits (100%) and 12 thoracic vertebrae in all 138 rabbits (100%). In 42 rabbits, radiographs lacked part of the cervical spine and number of cervical vertebrae was not possible to count. Three out of 138 rabbits had six lumbar vertebrae (2.2%), and the remaining had seven lumbar vertebrae (97.8%). More variation was observed in number of sacral vertebrae: 10 rabbits (7.1%) had five, three rabbits (2.1%) had three, and the remaining 90.7% had four sacral vertebrae (*n* = 140). Examples of three sacral vertebrae (S3) are presented in Figs. 1C, amp and D and 3B. All caudal vertebrae were adequately visible in 44/47 rabbits of subpopulation 2. Number of caudal vertebrae was 14 in five rabbits (11.4%), 15 in 26 rabbits (59.1%), and 16 in 13 rabbits (29.5%) (*n* = 44). Median length of the last three lumbar vertebrae in 138 rabbits was L5 16.1 mm (range 13.6–17.5 mm), L6 15.4 mm (range 12.9–17.0 mm), and L7 12.5 mm (range 10.1–14.1 mm). Median length of the sacrum was 36.1 mm (range 23.3–44.4 mm) in 140 rabbits. Only L5 length was significantly shorter in juvenile rabbits compared to adults (*P* = 0.002, 15.8 mm and 16.3 mm respectively).

**Fig. 3:**
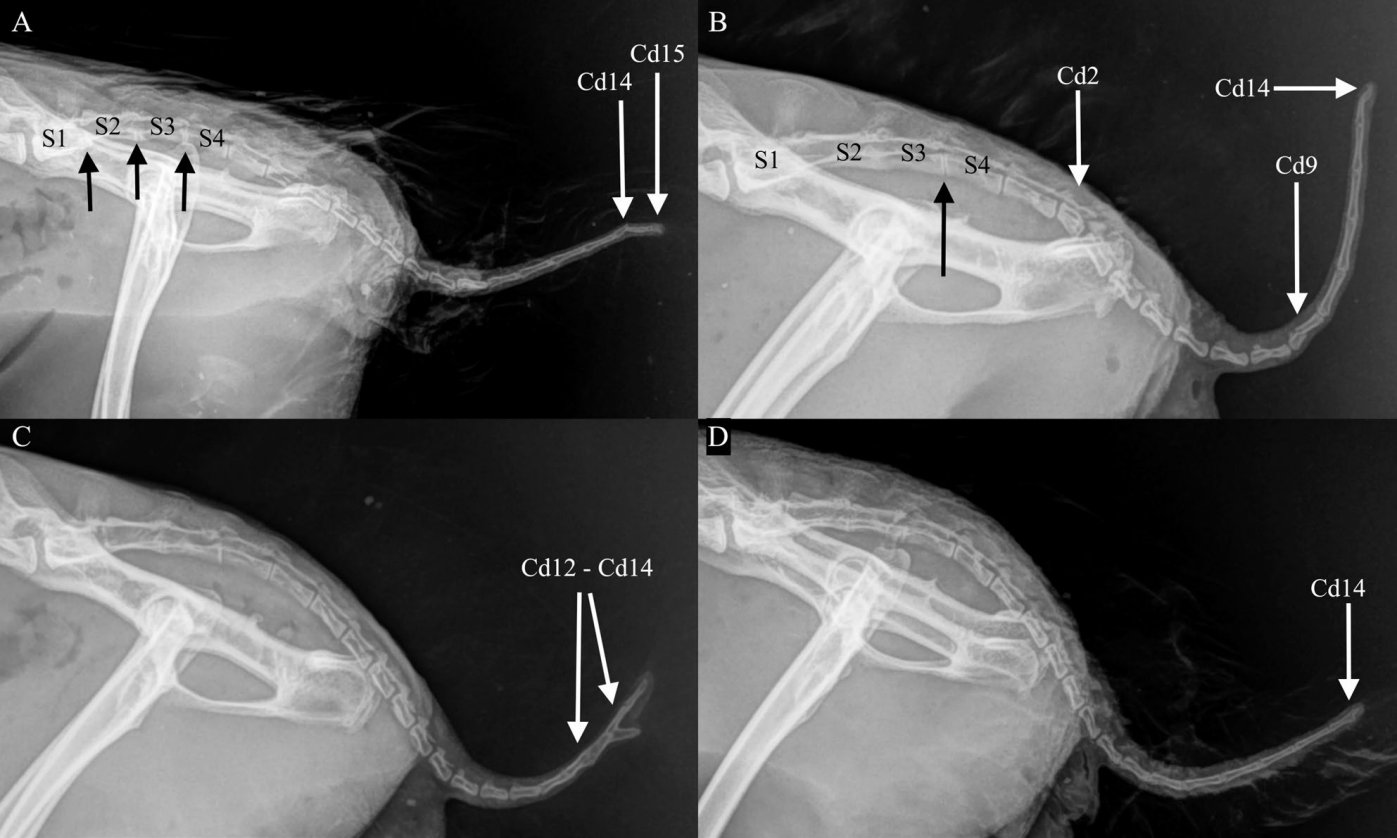
Examples of tail traumas and deformations in a population of Finnish wild rabbits. *Cd1* caudal vertebrae, *S* sacral vertebrae. **A**–**D** Laterolateral radiographs of the sacrum and tail of wild rabbits. **A** Unfused sacral vertebrae (vertebral spaces marked with black arrows). Fractures in Cd14 and Cd15 (marked with white arrows). **B** Only three sacral vertebrae when the S4 is transitional vertebra with the morphology of Cd1(S3-S4 vertebral space marked with black arrow). Cd2 is a hemivertebra (marked with white arrow). Fracture in Cd9 and deformed Cd14 (marked with white arrows). **C** Old fracture in Cd12-Cd14 healed in malunion (marked with white arrows). **D** Old fracture in Cd14 healed in malunion (marked with white arrow)

Out of 138 rabbits, location of the anticlinal vertebra was T11 in all but one rabbit, in which it was T12. This rabbit had 12 thoracic vertebrae and 7 lumbar vertebrae. The number of cervical vertebrae in this rabbit was not available. In one rabbit, the last thoracic vertebra (T12) was a transitional vertebra (unilateral rudimentary rib) in T12 and in one rabbit the first lumbar vertebra (L1) was a transitional vertebra (unilateral rudimentary rib). None of the rabbits had ribs at C7 (*n* = 129). The last lumbar vertebra (L7) was symmetrical in all rabbits (*n* = 47, 100%), as was the S1 (*n* = 47, 100%) and iliosacral joints (*n* = 45, 100%). One rabbit had an anomaly in two tail vertebrae as presented in Fig. 3B. None of the rabbits had cervical, thoracic or lumbar hemivertebrae or block vertebrae.

**Fig. 4:**
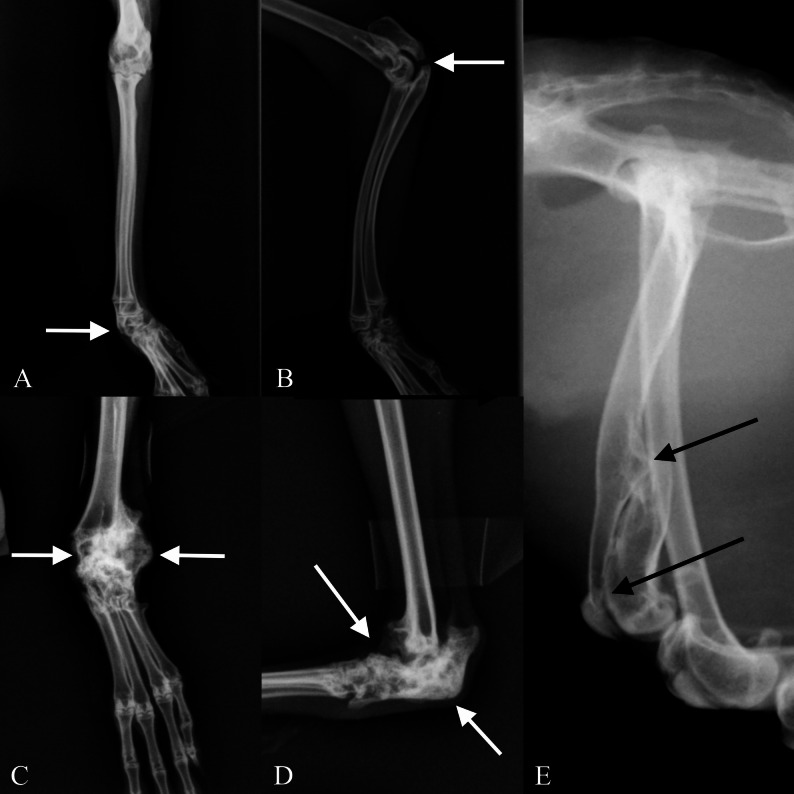
Examples of old extremity traumas in a population of Finnish wild rabbits. **A**, **B** Laterolateral and craniocaudal radiographs of the radius and ulna of a wild rabbit with carpal luxation and intra-articular non-union fracture of the proximal ulna (marked with white arrows). **C**, **D** Laterolateral and craniocaudal radiographs of the tarsus of a wild rabbit with old tarsal fracture and luxation that has led to synostosis of tarsal bones and tarsal valgus malunion (marked with white arrows). **E** Laterolateral radiograph of a wild rabbit femur with an old fracture healed in malunion (marked with black arrows)

Traumatic lesions were identified in radiographs of 21 rabbits (15%), with 13 rabbits having lesions in the vertebral column, mainly in the tail (*n* = 8). Examples of tail traumas are presented in Fig. 3A-D. Tail traumas were more common in male rabbits (*n* = 7) than in female rabbits (*n* = 1). Fractures of extremities or ribs were diagnosed in 11 rabbits (in total 13 injuries). Examples of fractures of extremities are presented in Fig. 4. Some rabbits (*n* = 3) had injuries in both the vertebral column and extremities or ribs. Osteoarthritis was found in four rabbits (5.0%), related to obvious intra-articular trauma in all of these cases. Additionally, spondylosis deformans was detected in five (3.6%) out of 140 rabbits.

It was possible to estimate closure of the physes from the radiographs of 90 rabbits. Of those, 36 rabbits (40%) had at least one visible radiolucent open physis (juvenile rabbits), 17 (18.9%) had at least some visible radio-opaque physeal scar (young rabbits), and 37 (41.1%) had all visible physes closed (adult rabbits) in the humerus, radius, ulna, distal femur, or tibia. Adult to younger (juvenile and young) rabbit ratio was 1:1.43 in this population.

## Discussion

Initial signs of dental disease (elongation of premolar tooth roots) were observed in the radiographs of 12.4% of the rabbits, but only two rabbits had mild abnormalities in oral examination of molars. These two rabbits did not, however, have signs of dental disease in skull radiograph. These mild irregularities in the molars might have been traumatic (e.g. caused by biting of hard substances) as they did not change the dental zig-zag pattern seen in the radiograph. Consistent with our results, Okuda et al. [11] reported a prevalence of 6.9% for elongation of the first premolar tooth and 10.3% for elongation of the first molar tooth root in wild hares. Our observed radiographic prevalence of dental disease is lower than the prevalence observed in the radiographs of Finnish pet rabbits (40%) [2]. Due to the progressive nature of dental disease, it is more common in older rabbits than in 1–3-year-old rabbits, and more advanced stages (grades 3–5) are usually diagnosed in rabbits over 3 years old [2, 32]. Exact age of the rabbits in our study was unknown as age estimation was made roughly from physes. Based on the closure of the physes, majority of the rabbits were, however, juvenile or young adults, which may explain the observed lower prevalence of dental disease. Reported mean age of wild rabbits is far less than three years so it is possible that older rabbits were lacking in our study [33, 34]. Only about 5–6% of newborn rabbits survive to the first reproductive season, and between the first two seasons the mortality is 40% [33]. The most common reasons for mortality in wild rabbits are diarrhoea due to coccidiosis [33], rabbit haemorrhagic virus (RHD), other diseases, predators, and accidents [34]. Cause of death in Finnish wild rabbits has not been studied, but the newly spread RHD (2016) and myxomatosis (2020) viruses have dramatically reduced the Finnish wild rabbit population [35]. The number of yearly hunted rabbits in Finland has dropped from 5000 (2015) to 115 (2023) [36]. Active culling projects, living in urban areas, traffic, and predators like urban foxes and eagle owls may also lower the mean age of wild rabbits in the Helsinki City area.

Energy-dense, low-calcium, and high-sugar/starch diets have been suggested to predispose to dental disease [6]. The diet of wild rabbits with a high fibre content and no commercial, high-energy, or high-carbohydrate food is considered ideal for pet rabbits as well. Rabbits living in the urban area of Helsinki City find their diet partly from parks, gardens, and allotment plots [9]. The diet of wild rabbits in Finland varies depending on the season, consisting mainly of hay and grass species during summer and twigs, buds, bark, needles, and sprouts of woody plants duringwinter, which have a high calcium concentration [9, 37]. During winter the selection of plants is restricted due to snow coverage and rabbits exploit all available fresh plants of urban areas such as flowers in cemeteries [9]. Carbohydrate and protein values of grass are lower in winter than in summer [33]. During the winter, the diet of wild rabbits seems to be of low energy density and high in calcium and low in carbohydrates, which should be optimal for preventing dental disease. Rabbits as selective and opportunistic eaters will select the most preferable parts of the diet, when possible, even though they have a predilection for fibres. Food preference is transmitted from does during gestation, nesting, and nursing periods [38]. Living area affects the seasonal and spatial variation of rabbits’ diets. In Portugal, cereals are an important part of wild rabbits’ diets, and acorns are consumed especially during the wintertime [8]. Allotment gardens are preferred by Finnish rabbits and provide a selection of fruits, berries, and vegetables with high carbohydrate concentrations during summer and autumn. Rabbits are also reported to eat dropped food from bird feeders in the Helsinki City area during winter. It is therefore possible that some rabbits, at least for part of the year, have more energy-dense and high-sugar diets. It is also possible that in urban areas the diet of wild rabbits has undergone changes through the generations towards a less optimal diet, and this could partly explain the observed prevalence of dental disease in this study.

Previously, low vitamin D concentrations were observed in Finnish wild rabbits [39]. Vitamin D deficiency has been suggested to be a predisposing factor for dental disease, but thus far, this relation has not been proven. Instead, several studies have reported no association between dental disease and UVB exposure, or 25-hydroxyvitamin D concentration [3, 40, 41]. Also in degus, UVB exposure had no effect on development of dental disease [42, 43]. In our study, only initial stage of dental disease was observed in a low number of Finnish wild rabbits, which does not give support for the suggested hypothesis of association between dental disease and vitamin D deficiency. Vitamin D does however have many other important functions, which have been rarely studied in rabbits. It has immunomodulant, anti-inflammatory, and anti-infective roles, and its deficiency has been linked to many chronic diseases and even to increased mortality in humans [44]. Further studies are needed to evaluate the effects of low vitamin D concentration on health of wild rabbits.

We observed mostly mild LTVs in wild rabbits and could not confirm any asymmetrical LTVs due to the lack of ventrodorsal radiographs, while in domestic rabbits the proportion of asymmetrical LTVs has been reported to be 1.8% [13]. In addition, we did not detect any asymmetrical abnormalities of L7 transitional processes on ventrodorsal or laterolateral radiographs. Almost all rabbits in our study population (97%) had 26 presacral vertebrae, as expected [12]. The anticlinal vertebra was T11 also in almost all wild rabbits, while in domestic rabbits the anticlinal vertebra has been much more variable [13]. The most common formula was the same C7/Th12/L7/S4 in our study population of wild rabbits (89.1%) as has been reported in domestic rabbits (76.3%) [13]. Thus, this formula can be considered normal. As previously described [13], we also observed several different vertebrae formulae in wild rabbits. However, the prevalence rates of these vertebral formulae were very low (0.7–3.6%) in our study population and are classified as different forms of LTVs based on previous literature [18, 30, 45].

None of the wild rabbits had ankylosing vertebral column deformities (hemivertebrae). Previously published prevalence rates of ankylosing vertebral column deformities in Finnish pet rabbits have been 18% in all pet rabbits and 40% in Dwarf Lop rabbits [2]. An overall prevalence of 15.2% for all congenital spinal abnormalities has been reported in a study of 330 domestic rabbits in Czech Republic [13]. The normal spinal morphology is important and thus evolutionarily conserved in rabbits, being fast dorsomobile runners whose spine flexes and extends rapidly while running and leaping [14, 18]. Thus, natural selection offers a possible explanation for the comparatively low prevalence of these congenital vertebral anomalies (hemivertebrae and transitional vertebrae) in wild rabbits despite originating from feral domesticated rabbits [35]. Abnormal standing position due to small cage size and heavy weight of the uterus during pregnancy with increased calcium demand during pregnancy and nursing leading to osteomalacia were suggested to predispose to ankylosing vertebral column deformities in breeding does [28]. However, as prevalence of ankylosing vertebral deformities has been reported to be higher in male pet rabbits (9.4%) than in females (6.0%) [13], this is a less probable explanation for ankylosing deformities in pet rabbits. In dogs, congenital hereditary vertebral anomalies are common [24, 46, 47] and clearly inheritable [25–27]. As higher prevalence was published in Dwarf Lop rabbits than in other breeds [2, 13], these deformities are most likely hereditary and congenital also in rabbits and should be taken into consideration in rabbit breeding.

Traumatic lesions were diagnosed in 15% of the rabbits. The most common site for injury was the tail, which could be caused by rabbits being chased by other rabbits or urban prey animals. Wounds and tail injuries were most common in male rabbits. This correlates with the observations of higher numbers of wounds in male rabbits than in female rabbits [33].

The wild rabbit population in Finland originates from pet rabbits released to nature mainly in the 1980s but also in the 2000s in the greater Helsinki region; the species was never released in the wild with the permission of the Finnish game authorities. The population is not therefore a ‘pure’ wild rabbit population but instead an urban feral rabbit population. Genetic diversity of Helsinki area rabbits has been studied by Laiho, and significantly lower than expected heterozygosity has been observed relative to other wild rabbit populations, in fact even lower than in domestic rabbit breeds [35]. Surprisingly, heterozygosity was significantly higher in the rabbit population of 2019–2020 than in the population of 2008–2009 despite RDH and myxomatosis epidemic bottlenecks in 2016 and 2020 [35]. The most probable explanation for this was the release of pet rabbits into the wild, as unusual colour variations and other domestication features in the fur were found in 15% of the rabbits sampled in her study [35]. Therefore, high gene flow from escaped domestic rabbits might explain also partly the common pet rabbit disorders found in this population. Replicating this study in a less urban wild rabbit population with known genetic diversity would be a logical next step.

This study has some limitations that should be addressed. Diagnosis of mild dental disease in these rabbits was made based on elongation of the premolar tooth roots in lateral skull radiographs, which is not a very sensitive method. The cut-off between normal variation and very early disease is ambivalent, and it is therefore possible that some healthy rabbits have been diagnosed as diseased, and vice versa. Computed tomography imaging is a more precise diagnostic tool but was not employed in this study for financial and availability reasons. Ventrodorsal radiographs were available only for 47 rabbits, limiting the analysis on lateral hemivertebrae, rudimentary contralateral rib and vertebral and sacral symmetry. In addition, indirect digital radiography with somewhat poorer image quality was used for 93 rabbits which may have reduced the detection of some minor abnormalities. Three assessors evaluated the radiographs, but we did not address the interrater repeatability in grading dental and skeletal abnormalities in rabbits, on which future studies are warranted. Post-mortem examination was not performed, and the full health status of the rabbits was therefore unknown. Based on previous studies on mortality of wild rabbits, theprevalence of diseases is high, especially in the winter [33]. Diseases may have influenced the overall welfare of rabbits, caused malnutrition, and affected sample size by increasing the number of young and diseased animals. None of the animals were, however, emaciated or severely diseased on physical examination.

## Conclusions

Prevalence of common diseases of pet rabbits, that are related to genetics, diet, and housing, was low in wild rabbits. Initial stages of dental disease (mild elongation of first premolar tooth roots) were identified in about every eighth rabbit in this wild rabbit population. Normal vertebral formula C7/Th12/L7/S4 was predominant. Ankylosing deformities were lacking, but mild anomalies were found in vertebral column radiographs. Traumatic lesions were common.

## Data Availability

The datasets used and/or analysed during the current study are available from the corresponding author on reasonable request.

## Abbreviations

C: Cervical vertebrae
Cd: Caudal vertebrae
L: Lumbar vertebrae
LTV: Lumbosacral transitional vertebra
S: Sacral vertebrae
T: Thoracic vertebrae

## Declarations

### Prior publication

Data have not been published previously.
